# Development and Validation of an ICP-MS/MS Method for Multi-elemental Analysis of Human Hair with Application to Heart Failure

**DOI:** 10.1007/s12011-026-05217-z

**Published:** 2026-07-06

**Authors:** Pablo Ramírez-Castillo, Alba García-Suarez, Pedro Morillas-Blasco, Nuria Vicente-Ibarra, Antonio García-Honrubia, Marina Martínez-Moreno, Raquel Sánchez-Romero, Ana Beltrán-Sanahuja, José-Luis Todolí-Torró

**Affiliations:** 1 Department of Analytical Chemistry, Nutrition and Food Science, P.O. Box 99, Alicante, 03080 Spain; 2 Cardiology Service, Elche University Hospital, Alicante, Spain

**Keywords:** ICP-MS/MS, Hair analysis, Trace elements, Method validation, Heart failure, Biomarkers

## Abstract

Human hair has emerged as a promising non-invasive matrix for assessing long-term exposure to essential and toxic trace elements relevant to cardiovascular disease. However, its clinical applicability depends on the availability of robust and fully validated analytical methodologies capable of overcoming matrix-related challenges. This study aimed to develop and validate a reliable and high-throughput method for multi-elemental (As, Cd, Cu, Pb, Se and Zn) analysis of human hair using microwave-assisted acid digestion followed by ICP-MS/MS, and to explore its potential application in a pilot cohort of heart failure (HF) patients. Method validation was performed using the certified reference material ERM-DB001. Analytical performance was evaluated in both no-gas and He-KED modes, assessing precision, trueness, procedural detection limits, and measurement uncertainty according to EURACHEM/CITAC guidelines. The influence of sample mass (20–200 mg) was also investigated. The validated method was subsequently applied to hair samples from 25 HF patients and 25 control subjects. The method demonstrated satisfactory intra- and inter-day precision, particularly for Cd, Cu, Se, and Zn. He-KED mode effectively reduced polyatomic interferences, improving selectivity and lowering detection limits for elements such as As and Se. Optimal analytical performance was achieved for sample masses between 60 and 100 mg, while lower masses resulted in increased uncertainty. In the pilot application, exploratory differences were observed for Pb, Mg, Ca and Zn concentrations, together with changes in selected elemental correlations (Mg/Na, Pb/Mn, Sr/Ni, Fe/Al, Fe/Ti and Mn/Ti) between heart failure patients and controls. The validated ICP-MS/MS method provides a sensitive and reliable approach for multi-elemental analysis of human hair. Its robustness and applicability to real samples support its potential use in clinical and epidemiological studies, although further research with larger cohorts is required to confirm the observed trends.

**Supplementary Information:**

The online version contains supplementary material available at https://doi.org/10.1007/s12011-026-05217-z.

## Introduction

Heart failure (HF) remains one of the leading causes of morbidity and mortality worldwide, representing a major and growing public health challenge [1, 2]. In Spain, cardiovascular conditions account for a significant proportion of annual fatalities, ranking second only to cancer [3]. In addition to well-established clinical and metabolic risk factors, increasing attention has been directed toward the role of chronic exposure to essential and toxic trace elements in cardiovascular disease. Given that the management of HF frequently involves depletive or diuretic therapies aimed at regulating fluid volume, maintaining electrolyte homeostasis is a key aspect of patient care. These elements are involved in key biological processes, including oxidative stress regulation, inflammation, and cellular signaling, and their imbalance may contribute to the progression of cardiac dysfunction [1, 4]. In this context, the identification of reliable biomarkers capable of reflecting long-term exposure and metabolic status is of particular relevance for both clinical and epidemiological studies.

Among the available biological matrices, human hair has emerged as a promising non-invasive alternative for trace element analysis. Unlike blood or urine, hair provides a retrospective record of elemental incorporation over time, reflecting both endogenous metabolism and environmental exposure [5, 6]. However, its use as a clinical biomarker remains controversial [7] due to the complexity of the matrix, its susceptibility to external contamination, and the analytical challenges associated with the accurate quantification of elements at trace and ultra-trace levels [8, 9]. These limitations highlight the need for highly sensitive, selective, and well-validated analytical methodologies capable of ensuring data reliability and reproducibility.

Inductively coupled plasma tandem mass spectrometry (ICP-MS/MS) has become a state-of-the-art technique for multi-elemental analysis, offering significant advantages in terms of sensitivity, selectivity, and interference control [10, 11]. The use of collision/reaction cell technology, particularly in He-KED mode, allows for effective removal of polyatomic interferences [12, 13], which are especially relevant in complex matrices such as hair. In combination with optimized sample preparation procedures, including microwave-assisted acid digestion, ICP-MS/MS enables accurate quantification of a wide range of elements at ultra-trace concentrations. Despite these advantages, there is still a limited number of fully validated methodologies specifically adapted to hair analysis in a clinical context, and few studies have explored its application in cardiovascular disease [14, 15].

Consequently, the aim of this study was to develop and validate a robust methodology using microwave-assisted acid digestion and ICP-MS/MS for multi-elemental analysis. This validated method was subsequently applied to a pilot clinical cohort with the specific objective of evaluating whether the measurements of certain ions in the hair of heart failure patients undergoing diuretic or depletive treatment differ significantly from those of control subjects.

## Materials and Methods

### Reference Material and Hair Samples

The validation of the analytical method using ICP-MS/MS was carried out with the certified reference material ERM-DB001 (IRMM, Geel, Belgium), which provides certified values for As, Cd, Cu, Hg, Pb, Se, and Zn, as well as informational values for 34 trace elements and MeHg [16].

Human hair samples were provided by the Cardiology Service of the Elche University Hospital (Alicante, Spain). A total of 25 patients diagnosed with heart failure with reduced ejection fraction (< 50%) were included, along with another 25 patients without cardiovascular disease who served as the control group.

The samples were collected from the occipital region of the head and placed in sterile paper-plastic self-sealing bags to ensure proper transport and preservation until analysis. The whole available hair sample was subjected to washing of the samples was carried out following a procedure adapted from the cleaning protocol recommended by the IAEA [17], with the aim of removing external contaminants without altering the internal trace element content. For this purpose, the samples underwent three consecutive washes with acetone, followed by three 5-minute washes with deionized water, and finally three additional 5-minute washes with acetone, maintaining constant agitation throughout all stages. After the final wash, the residual acetone was allowed to evaporate, and the samples were stored in the cleaning vessels until their acid digestion.

### Reagents and Solutions

All reagents used were of ultrapure grade. The digestion process was performed using nitric acid (HNO_3_, 69%, Ultratrace^®^, Scharlab, Barcelona, España), suitable for trace-level metal analysis. Ultrapure water (resistivity 18.2 MΩ·cm, Milli-Q^®^ system, Millipore, United States) was used for both solution preparation and sample washing.

Calibration curves were prepared by diluting a 100 mg kg^-1^ multielement stock solution (SCP33MS) purchased from SCP SCIENCE (Clark-Graham, Baie-D’Urfé, Canada). The calibration range covered concentrations from 0.1 to 1000 µg kg^-1^, depending on the element.

To correct for potential instrumental variations and matrix effects, an internal standard solution containing scandium (^45^Sc), germanium (^72^Ge), rhodium (^103^Rh), and rhenium (^185^Re) was used, also purchased from SCP SCIENCE. These standards were introduced in line with the sample stream through a continuous mixing system, maintaining a final concentration of 40 µg kg^-1^.

### Sample Digestion Procedure

The samples were mineralized by microwave-assisted acid digestion using 8 mL of ultrapure HNO_3_ in an ETHOS TOUCH CONTROL system (Milestone, Sorisole, Italy).

For method validation with the certified reference material (ERM-DB001) [16], 200 mg of sample were weighed, following the manufacturer’s recommendations.

The precision of the method was evaluated through the analysis of the certified reference material ERM-DB001 over five different days, with three independent digestions performed each day. Repeatability (intra-day precision) was calculated as the relative standard deviation (RSD) of the triplicates within the same day, while intermediate precision (inter-day) was expressed as the RSD of the daily means across the five days.

Procedural limits of detection (pLOD) and quantification (pLOQ) were calculated for each certified element using the standard deviation of procedural blanks and the slope of the calibration curve, following the IUPAC approach for detection and quantification capabilities [18]. In this study, the limits were expressed on a procedural basis by considering the complete sample preparation procedure, including the final mass of the digest and the initial sample mass. These parameters were derived from the standard deviation of procedural blanks ($\:{\sigma\:}_{blank}$), the slope of the calibration curve (* s*), the final mass of the digest ($\:{m}_{f}=12\:g$) and the mass of the sample ($\:{m}_{s}=0.2\:g$), according to the following expressions:

1 $$pLOD=\frac{3{\sigma\:}_{blank}\cdot m_f}{s\cdot m_s}$$

2 $$pLOQ=\frac{10{\sigma\:}_{blank}\cdot m_f}{s\cdot m_s}$$

In the study of mass influence, different amounts were analyzed. In addition to the 200 mg used for method validation, 100, 60, and 20 mg were also weighed, while keeping the acid volume, digestion program, and instrumental conditions constant, in order to assess the effect of initial mass on method accuracy and precision. For real samples, the amount of hair used depended on the material available.

The Spearman correlation analysis was performed using Jamovi [19] (version 2.6.44). All graphical representations, including the correlation plots, were generated using Microsoft Excel, facilitating the visualization and interpretation of the results.

The digestion program consisted of a progressive heating up to 200 °C over 15 min, followed by a 15-minute hold at this temperature, with a maximum power of 1500 W.

Between each batch of samples, an intermediate digestion using only nitric acid was performed to prevent cross-contamination between vessels, and every ten digestions a procedural blank was included and subjected to the same treatment to verify potential contamination from the reagent or the system.

After digestion, the resulting solutions were cooled to room temperature and brought to a final mass of 12 g with ultrapure water. The solutions were stored at 4 °C until their analysis by ICP-MS/MS.

Post-digestion spike recovery experiments were performed to evaluate the accuracy of the quantification step and possible matrix effects in the digested hair solutions. For this purpose, aliquots of previously digested ERM-DB001 solutions were fortified with the multielement standard solution to obtain final added concentrations of 5 and 200 µg kg^-1^ in the analytical solution. The spiked and non-spiked digests were then analyzed under both no-gas and He-KED modes.

### ICP-MS/MS Instrumentation and Operating Conditions

The analysis was performed using an Agilent 8900 tandem inductively coupled plasma mass spectrometer (ICP-MS/MS) (Agilent Technologies, California, United States), equipped with an Ultra High Matrix Introduction (UHMI) system that enables the handling of high-ionic-strength matrices, and a collision cell operated with helium (He) as collision gas under kinetic energy discrimination (KED) mode, also operable in no-gas mode. This component helped to compensate for spectral interferences caused by the hair matrix (Table S1). The plasma operating conditions were kept constant throughout the validation (Table 1).

**Table 1:** Operating conditions of the ICP-MS/MS in no-gas and He-KED modes

Parameter	No-gas mode	He-KED mode
Radiofrequency power (W)	1550	1550
Carrier gas (L min^− 1^)	1.09	1.09
He flow (mL min^− 1^)	0	5.0
Energy discrimination voltage (V)	5.0	3.0
Sample uptake rate (mL min^− 1^)	0.3	0.3
Sampling depth (mm)	10	10
Sweeps	100	100
Replicates	3	3
Dwell time (s)	0.3	0.3
Spray chamber temperature (°C)	2	2
Selected isotopes	^23^Na, ^24^Mg, ^27^Al, ^39^K, ^43^Ca, ^47^Ti, ^51^V, ^52^Cr, ^55^Mn, ^57^Fe, ^59^Co, ^60^Ni, ^65^Cu, ^66^Zn, ^75^As, ^78^Se, ^85^Rb, ^88^Sr, ^95^Mo, ^107^Ag, ^111^Cd, ^118^Sn, ^137^Ba, ^139^La, ^140^Ce	^23^Na, ^24^Mg, ^27^Al, ^39^K, ^43^Ca, ^47^Ti, ^51^V, ^52^Cr, ^55^Mn, ^57^Fe, ^59^Co, ^60^Ni, ^65^Cu, ^66^Zn, ^75^As, ^78^Se, ^85^Rb, ^88^Sr, ^95^Mo, ^107^Ag, ^111^Cd, ^118^Sn, ^137^Ba, ^139^La, ^140^Ce, ^208^Pb

### Uncertainty Calculation

The estimation of the uncertainty associated with the analytical procedure was carried out in accordance with the guidelines established in the EURACHEM/CITAC guide *“Quantifying Uncertainty in Analytical Measurement”* [20] and the principles of ISO/IEC 17025:2017 [21], applying a statistical approach based on the propagation of normalized errors.

The uncertainty model adopted included the main contributions of the analytical process: calibration uncertainty ($\:{u}_{cal}$), repeatability uncertainty ($\:{u}_{r}$), intermediate precision uncertainty ($\:{u}_{ip}$), and trueness uncertainty ($\:{u}_{t}$). Detailed calculations of the individual uncertainty contributions, as well as the combined and expanded uncertainties are provided in Text S1.

All components were expressed as relative percentages with respect to the measured analyte concentration, ensuring dimensional consistency prior to their combination.

## Results

### Method Validation

The analytical performance of the ICP-MS/MS method was evaluated using the certified reference material ERM-DB001. As shown in Table 2, the method demonstrated good intra- and inter-day precision for all certified elements. The lowest variability was observed for Cd, Cu, Se, and Zn. Higher RSD values were obtained for As, particularly under no-gas conditions, which is consistent with the stronger influence of polyatomic interferences on this element. A relatively high intra-day RSD was also observed for Pb in both acquisition modes. However, instrumental repeatability for Pb, estimated from replicate readings of individual digest solutions, was acceptable in both no-gas and He-KED modes, with mean RSD values of 1.7% and 0.6%, respectively. Therefore, the higher intra-day RSD observed for Pb was mainly attributed to procedural variability, including independent digestion, sample weighing, and possible small-scale heterogeneity of the reference material, rather than to instrumental repeatability.

**Table 2:** Intra-day and inter-day relative standard deviation for certified elements under no-gas and He-KED modes

	As	Cd	Cu	Pb	Se	Zn
Intra-day RSD No Gas (%)	12.8	3.8	1.6	16.7	2.7	0.8
Intra-day RSD He-KED (%)	11.2	4.1	1.6	16.6	2.5	0.8
Inter-day RSD No Gas (%)	32.6	3.6	5.1	4.7	6.0	3.1
Inter-day RSD He-KED (%)	20.7	2.6	3.9	5.5	3.0	3.2

The use of He-KED mode led to a clear improvement in precision for elements affected by polyatomic interferences (Table S1). For example, the inter-day RSD for arsenic decreased from 32.6% in no-gas mode to 20.7% in He-KED mode. In contrast, elements less affected by spectral interferences, such as Zn and Pb, showed similar RSD values in both modes.

Procedural limits of detection (pLOD) and quantification (pLOQ) are presented in Table 3. Lower pLOD values were obtained in He-KED mode for elements such as As and Se, confirming the effectiveness of helium in reducing spectral interferences. Table S2 illustrates the impact of the He flow rate on the Se signal decrease for a digestion blank. For elements such as Cd, Cu, and Zn, no significant differences were observed between modes.

**Table 3:** Procedural LOD and LOQ for certified elements in no-gas and He-KED modes

	As (mg kg^− 1^)	Cd (mg kg^− 1^)	Cu (mg kg^− 1^)	Pb (mg kg^− 1^)	Se (mg kg^− 1^)	Zn (mg kg^− 1^)
pLOD no gas	0.03	0.006	0.009	0.005	0.3	0.08
pLOD He-KED	0.009	0.007	0.01	0.006	0.03	0.12
pLOQ no gas	0.09	0.02	0.03	0.02	1.1	0.3
pLOQ He-KED	0.03	0.02	0.03	0.02	0.1	0.4

The comparison between measured and certified values (Table 4) showed acceptable trueness for most certified elements, although some element-specific deviations were observed. As, Cd, and Se showed good agreement with the certified values, whereas Pb showed a positive bias in both acquisition modes (126.2%) and Zn showed a slight negative bias (78.5–78.9%). The high Pb recovery may be partly related to residual matrix effects and possible differences in Pb isotopic composition between the natural-abundance calibration standard and the hair reference material. In contrast, the slightly low recovery obtained for Zn suggests a moderate negative bias, probably associated with matrix effects affecting signal transmission or internal standard correction. Therefore, Zn results were interpreted considering this slight bias, particularly given its relevance in the context of cardiovascular disease and heart failure.

**Table 4:** Measured concentrations, confidence intervals and recoveries compared with certified values*

	As	Cd	Cu	Pb	Se	Zn
Certified value ± U	0.044 ± 0.006	0.125 ± 0.007	33 ± 4	2.14 ± 0.20	3.24 ± 0.24	209 ± 12
No gas concentration ± CI (mg kg^− 1^)	0.048 ± 0.010	0.141 ± 0.004	27.7 ± 0.6	2.7 ± 0.3	3.32 ± 0.10	165 ± 2
He-KED concentration ± CI (mg kg^− 1^)	0.050 ± 0.006	0.110 ± 0.003	40.0 ± 0.7	2.7 ± 0.3	3.10 ± 0.08	164 ± 2
No gas recovery (%)	109.1	112.8	83.9	126.2	102.5	78.9
He-KED recovery (%)	113.6	88.0	121.2	126.2	95.7	78.5

*Certified values are reported with their expanded uncertainties (k = 2), as stated in the ERM-DB001 certificate. Measured values are shown with their 95% confidence intervals (CI), calculated as$\:\frac{t\:s}{\sqrt{n}}$with *n* = 15 and t = 2.145 (for 14 degrees of freedom). Recoveries were calculated as (measured concentration / certified value) × 100

Post-digestion spike recoveries were evaluated for 25 measured elements at two added concentration levels, equivalent to 5 and 200 µg kg-^1^ in the analytical solution, in both no-gas and He-KED modes (Table S3). Most recoveries were within the 70–130% range.

### Interference Evaluation

Spectral interferences were identified as a critical factor affecting analytical accuracy. Selenium was selected as a model element due to its susceptibility to polyatomic interferences. Optimization of helium flow showed that a value of 5 mL min^-1^ was sufficient to effectively reduce these interferences.

Although operation in He-KED mode reduced signal intensity for some elements, it improved selectivity and signal reliability. In some cases, signal enhancement was observed, likely due to improved ion transmission efficiency within the collision cell (Figure S1).

Non-spectral matrix effects were evaluated using the internal standard signals. As shown in Figure S2, the internal standard signals in digested hair samples were higher than those obtained in aqueous calibration standards, with increases close to two-fold for some internal standards. This behavior indicated a significant matrix effect, probably associated with residual dissolved carbon and other matrix constituents present in the digested hair solutions. These effects were corrected for by continuous online addition of internal standards and normalization of the analyte signals.

The possible contribution of carbon-induced signal enhancement was also considered. Carbon-containing matrices are known to enhance the ionization of hard-to-ionize elements in ICP-MS, particularly As and Se, through changes in plasma conditions and charge-transfer reactions involving carbon-containing species [22]. Therefore, the enhanced signals observed in digested hair samples may partly reflect this phenomenon. Since calibration standards and digested samples may differ in residual carbon content, this effect represents a potential source of bias if not properly corrected. In the present study, internal standard correction, validation with ERM-DB001, and post-digestion spike recovery experiments were used to verify that the matrix correction was adequate under the selected operating conditions. Nevertheless, matrix-matched calibration or standard addition could be considered in future applications to further reduce matrix-related bias and improve the comparability between aqueous calibration standards and digested hair samples.

### Recovery of Non-certified Elements

Recovery tests performed for non-certified elements (Table S3) showed consistent results at both evaluated spike levels (5 and 200 µg kg^-1^). For elements present at high natural concentrations, recovery values were not calculated due to the limited impact of spiking.

Overall, the results confirmed that the method provides reliable measurements across a wide range of elements.

### Influence of Sample Mass

The effect of sample mass on analytical uncertainty is shown in Table 5. The lowest uncertainties were obtained for sample masses between 60 and 100 mg, whereas a significant increase in uncertainty was observed at 20 mg.

**Table 5:** Concentration values and expanded relative uncertainties at different sample masses. Expanded uncertainties were calculated using a coverage factor of k = 2

Element	Concentration, mg kg^− 1^ (Expanded relative uncertainty, %)			
	200 mg	100 mg	60 mg	20 mg
As	0.053 (33%)	0.052 (26%)	0.060 (35%)	0.060 (43%)
Cd	0.122 (11%)	0.138 (9.6%)	0.132 (7.3%)	0.151 (21%)
Cu	37 (12%)	39 (18%)	39 (13%)	43 (36%)
Se	3.10 (10%)	3.78 (8.9%)	3.59 (9.7%)	3.51 (11%)
Pb	2.42 (25%)	2.44 (22%)	2.43 (19%)	2.94 (28%)
Zn	235 (7.2%)	242 (7.3%)	240 (7.2%)	235 (10%)

This effect was particularly pronounced for elements present at low concentrations or affected by interferences, such as As and Cd. In contrast, Zn and Se showed relatively stable uncertainty across all tested masses. To facilitate visualization of this effect, recoveries relative to the certified values together with their expanded uncertainties are additionally provided in Figure S3.

### Application to Real Samples

**Fig. 1:**
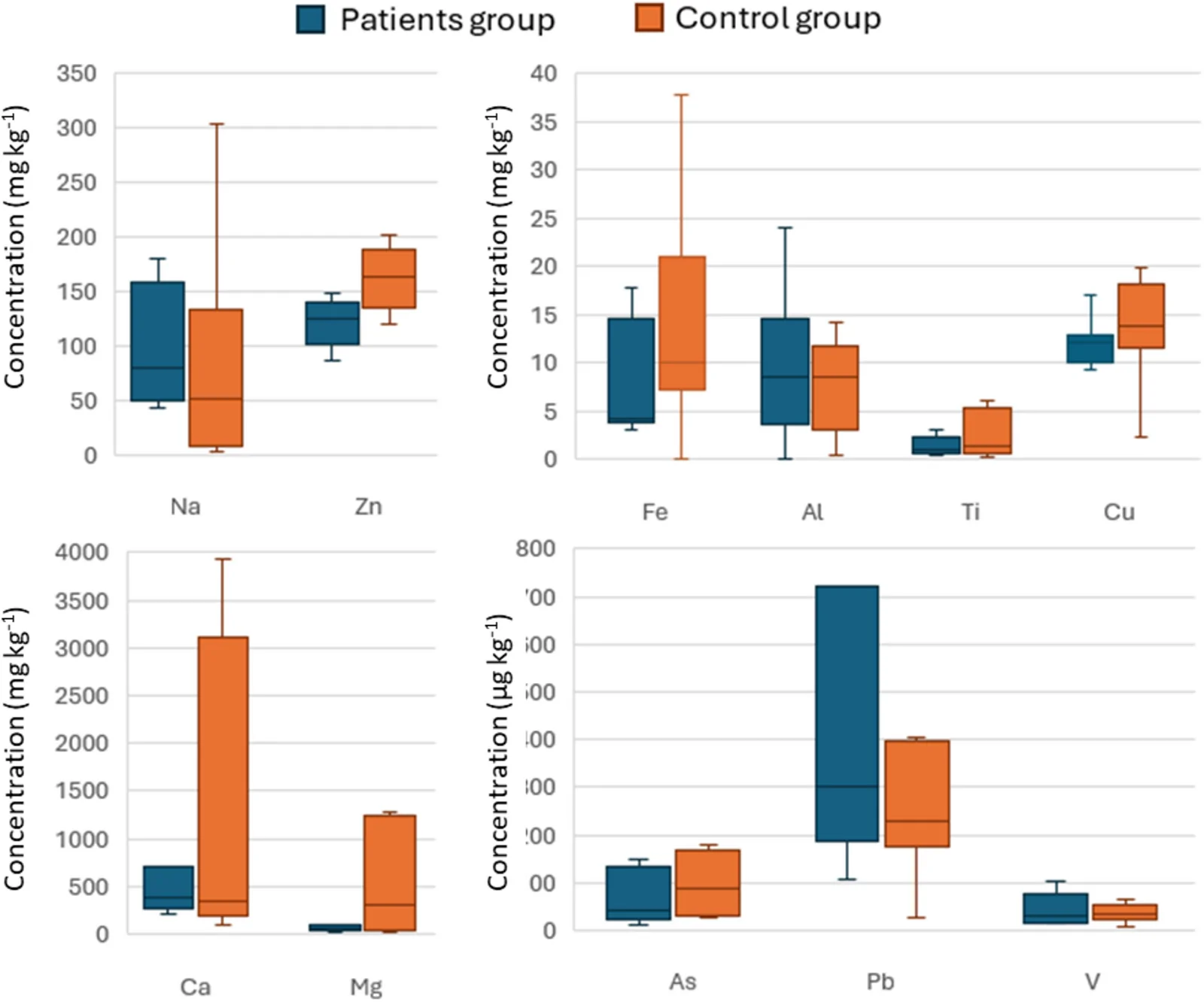
Concentrations of the elements present in hair for the patient group and the control group

The validated method was applied to hair samples from 25 heart failure patients and 25 control subjects. All elements included in the validated ICP-MS/MS method were determined in the real samples. Figure 1 shows a representative subset of elements selected because they exhibited the clearest between-group differences, higher dispersion, or greater clinical/environmental relevance in the context of heart failure and trace element imbalance.

Variability was observed in essential elements such as Zn, Ca, and Mg in both groups, whereas Pb showed higher concentrations and dispersion in the patient group. Other elements, including Fe, Ti, Al, and V, exhibited overlapping distributions.

**Fig. 2:**
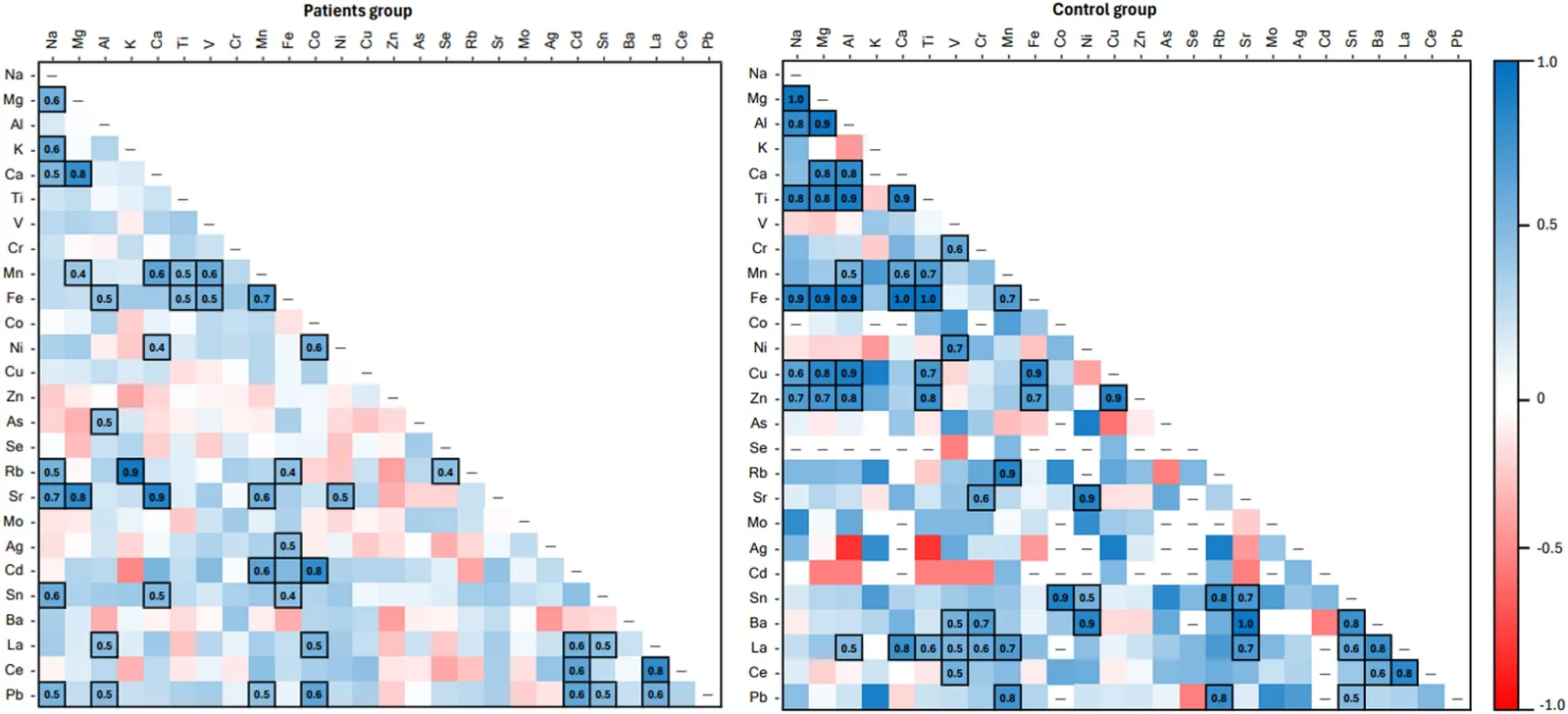
Spearman correlation matrix for the patients and control groups. Correlations with *p* < 0.05 are highlighted with black cell borders. Figures inside the boxes correspond to correlation coeficients.

Spearman correlation analysis (Fig. 2; Table 6) revealed differences in elemental relationships between groups, suggesting that the distribution of trace elements may be influenced by physiological or environmental factors.

**Table 6:** Spearman correlation values for pairs of elements showing significant differences between patients and controls (*p* < 0.05)

Element pair	Patients	Controls
Mg/Na	0.56	0.95
Pb/Mn	0.45	0.86
Sr/Ni	0.54	0.94
Fe/Al	0.46	0.96
Fe/Ti	0.48	0.70
Mn/Ti	0.49	0.95

## Discussion

The present study demonstrates that ICP-MS/MS, combined with microwave-assisted digestion, provides a robust and reliable analytical approach for multi-elemental analysis in human hair.

Human hair has emerged as a promising non-invasive matrix for assessing long-term exposure to essential and toxic trace elements, reflecting both endogenous metabolism and environmental exposure [5, 6]. However, its application remains analytically challenging due to matrix complexity and susceptibility to contamination [8, 9]. In this context, the validation results obtained in this study confirm that these limitations can be effectively addressed through appropriate sample preparation and instrumental optimization.

The improvement observed in precision and detection limits under He-KED mode highlights the importance of interference control in complex biological matrices. ICP-MS/MS has become a state-of-the-art technique for multi-elemental analysis due to its high sensitivity, selectivity, and ability to reduce polyatomic interferences [10]. The results obtained here confirm that helium collision gas plays a critical role in improving analytical performance, particularly for elements such as As and Se. The analytical performance observed in this study is in line with previous reports highlighting the advantages of ICP-MS/MS for multi-elemental analysis, particularly its ability to reduce spectral interferences and improve detection limits in complex matrices [10, 14].

The evaluation of sample mass demonstrated that analytical uncertainty is strongly dependent on the amount of material used. While masses between 60 and 100 mg provided optimal results, lower masses significantly increased uncertainty. This finding is particularly relevant in clinical applications, where sample availability may be limited. Since hair collection is non-invasive, the use of a larger sample mass can generally be considered when sufficient material is available. In addition to improving analytical uncertainty, a larger amount of hair may provide retrospective information on elemental exposure over extended periods.

The application of the validated method to real samples revealed differences in the distribution of 26 elements between heart failure patients and controls. Although these observations are exploratory, they suggest that trace element profiles in hair may reflect physiological alterations associated with cardiovascular disease. These findings are consistent with previous studies reporting alterations in trace element profiles in hair samples from patients with cardiovascular diseases, suggesting that hair may reflect systemic metabolic and environmental changes associated with these conditions [4, 6].

Trace elements are involved in key biological processes, including oxidative stress, inflammation, and cellular signaling, and their imbalance may contribute to cardiac dysfunction [1, 4]. The variability observed in elements such as Mg, Ca, and Zn [23] may be related to disease-specific mechanisms, including diuretic therapy and altered mineral metabolism. Some essential elements such as Zn, Ca, and Mg exhibited high dispersion in both groups, with particularly elevated maximum values in the control group. These variations are likely driven by the disease’s specific pathophysiological alterations, including the sequestration of Ca and Mg in inflamed tissues and atherosclerotic plaques, as well as the chronic use of loop diuretics, which promotes the systemic depletion of these ions through urinary wasting. Critically, these elemental imbalances have direct repercussions on myocardiocyte function and cardiac contractility [1, 24]. Magnesium acts as a natural calcium antagonist, regulating excitation-contraction coupling (ECC) and protecting cardiomyocytes from toxic calcium overload. Therefore, the low magnesium availability reflected in hair samples may indicate a potential association with impaired systolic function and increased susceptibility to life-threatening arrhythmias, which progressively worsen the clinical course of heart failure [24]. Similar trends have been described in earlier studies linking trace element imbalances with cardiovascular dysfunction, particularly in relation to oxidative stress and altered mineral metabolism [1, 24].

In addition, the observed differences in correlation patterns between elements suggest that not only absolute concentrations, but also elemental relationships, may be affected by pathological conditions.

In this regard, the correlation analysis provided additional information beyond individual elemental concentrations. The Mg/Na correlation may reflect differences in electrolyte and mineral homeostasis, which is particularly relevant in heart failure because these elements are closely linked to fluid balance, renal handling, and the use of diuretic therapy [25]. The Pb/Mn relationship may indicate an altered balance between toxic metal exposure and essential trace element status, since Mn participates in antioxidant defense and mitochondrial metabolism [26], whereas Pb is a non-essential toxic element associated with oxidative stress and cardiovascular risk [27]. Similarly, the correlations involving Fe may be relevant because iron metabolism is closely related to oxygen transport, mitochondrial function, and inflammatory status, all of which are important in cardiovascular disease [28]. Overall, these findings suggest that altered elemental relationships in hair may capture combined physiological and exposure-related patterns, although their clinical significance requires confirmation in larger cohorts.

Despite these promising findings, the study has several limitations. First, the elemental profile of hair may be influenced by diet, lifestyle, cosmetic treatments, and environmental exposure. Although the IAEA-based washing procedure was applied to reduce external contamination, complete discrimination between endogenous incorporation and exogenous deposition cannot be guaranteed, particularly for toxic elements such as Pb. Therefore, the observed differences should be interpreted as exploratory hair-based profiles reflecting both physiological and exposure-related contributions. The sample size is relatively small, and the clinical interpretation of the results should be considered exploratory. Further studies with larger cohorts are required to confirm these observations and to establish the clinical relevance of hair-based elemental analysis. These results also support previous observations indicating that, despite the analytical challenges associated with hair as a biological matrix, appropriate sample preparation and instrumental optimization can significantly improve data reliability [8, 9].

Overall, the results support the potential of validated ICP-MS/MS methodologies as reliable tools for trace element analysis in complex biological matrices, with promising applications in clinical and epidemiological research.

## Conclusions

This study presents a fully validated and methodologically rigorous approach for the multi-element analysis of human hair using microwave-assisted digestion and ICP-MS/MS. The results demonstrated that this analytical workflow provided the sensitivity, selectivity, and robustness required for clinical applications, particularly in the context of complex matrices such as keratinized tissues.

One of the key findings of the validation process was the necessity of employing He-KED mode for elements prone to extensive polyatomic interference, including arsenic and selenium. The collision cell significantly improved signal accuracy by reducing background and interference-derived noise, ultimately enhancing both detection limits and trueness.

The study showed that sample mass plays a significant role in the analytical outcome. While masses between 60 and 100 mg yielded the lowest uncertainties, smaller sample sizes introduced substantial error due to factors such as heterogeneity, weighing precision, and reduced signal-to-noise ratios. This finding is particularly relevant for clinical and epidemiological studies, where available hair mass may vary widely.

The pilot application of the method to real samples from patients with confirmed compensated heart failure disease and matched controls provided a preliminary demonstration of the method’s clinical utility. Differences in concentrations of toxic elements such as lead, as well as variations in essential element patterns and elemental ratios suggested that hair composition may reflect underlying physiological or exposure-related factors associated with cardiovascular disease. Although these observations are exploratory due to the limited sample size, they support the notion that hair-based elemental profiling could contribute valuable information in the assessment of cardiovascular risk.

## Supplementary Information

Below is the link to the electronic supplementary material.

Supplementary File 1 (DOCX 61.0 KB)

## Data Availability

No datasets were generated or analysed during the current study.
