# Supplementary material for: Development and Validation of an ICP-MS/MS Method for Multi-elemental Analysis of Human Hair with Application to Heart Failure
Source: Biol Trace Elem Res. 2026 Jul 6;204(10):7711–20. doi: 10.1007/s12011-026-05217-z (PMC13615901; doi:10.1007/s12011-026-05217-z)
Supplement: Supplementary file 1 [file 12011_2026_5217_MOESM1_ESM.docx]

**Supporting Information**

# Development and Validation of an ICP-MS/MS Method for Multi-Elemental Analysis of Human Hair with Application to Heart Failure

Pablo Ramírez-Castillo,^1^ Alba García-Suarez,^2^ Pedro Morillas-Blasco,^2^ Nuria Vicente-Ibarra,^2^ Antonio García-Honrubia,^2^ Marina Martínez-Moreno,^2^ Raquel Sánchez-Romero,^1^ Ana Beltrán-Sanahuja,^1^ José-Luis Todolí-Torró^1^*

^1^ Department of Analytical Chemistry, Nutrition and Food Science, P.O. Box 99, 03080, Alicante, Spain.

^2^ Cardiology Service, Elche University Hospital, Alicante, Spain.

* Corresponding author: José-Luis Todolí-Torró

Email: jose.todoli@gcloud.ua.es

**Text S1.** Detailed calculations of individual uncertainty contributions and of combined and expanded uncertainties

- Calibration uncertainty ($u_{cal}$)

This component comprises the purity of the primary standard, the preparation of the calibration solutions, and the linear regression of the calibration curve. The uncertainty associated with the regression was calculated using the following equation:

$u_{cal}=\frac{s}{m}\cdot\sqrt{\frac{1}{n}+\frac{(C-\bar{C})^{2}}{\sum(C_{i}-\bar{C})^{2}}}$	(S1)

where $s$ is the residual standard deviation of the regression, $m$ the slope, $n$ the number of calibration points, $C$ the analyte concentration in the sample, $C_{i}$ the concentrations of the standards, and $\bar{C}$ the average of $C_{i}$.

- Repeatability uncertainty ($u_{r}$)

It evaluates the variability of consecutive repetitions of the complete procedure under identical analytical conditions. It was determined by one-way ANOVA, considering intraday replicates. The repeatability standard deviation was estimated as:

$s_{rep}=\sqrt{MS_{within}}$	(S2)

and the relative repeatability uncertainty was calculated as:

$u_{r}=\frac{s_{rep}}{\sqrt{n_{rep}}}$	(S3)

where $MS_{within}$ is the intraday mean square and $n_{rep}$ the number of replicates.

- Intermediate-precision uncertainty ($u_{ip}$)

It represents the variability of the method when the procedure is repeated on different days. It was also calculated by ANOVA, using the between-day ($MS_{between}$) and intraday ($MS_{within}$) variances, according to:

$s_{ip}=\sqrt{\frac{MS_{between}-MS_{within}}{n_{rep}}}$	(S4)

$u_{ip}=\frac{s_{ip}}{\sqrt{n_{days}}}$	(S5)

where $n_{rep}$ is the number of replicates per day and $n_{days}$ the number of analysis days.

- Trueness uncertainty ($u_{t}$)

This component reflects the contribution of the bias between the experimental results and the certified values of the reference material. Prior to this, the absence of significant bias was verified by applying the criterion:

$\mid\Delta_{m}\mid\leq t\cdot u_{\Delta}$	(S6)

where $\Delta_{m}$ is the absolute difference between the measured mean value and the certified value, $t$ is the Student’s $t$ value corresponding to a 95% confidence level, and $u_{\Delta}$ is the combined uncertainty associated with this difference:

$u_{\Delta}=\sqrt{u_{r}^{2}+u_{ip}^{2}+u_{CRM}^{2}}$	(S7)

where $u_{CRM}$ is the standard uncertainty associated with the certified reference material, calculated from the expanded uncertainty reported for the certified reference material (CRM) [12] ($U_{CRM}, k=2)$

Once it was confirmed that the bias was not significant, the relative trueness uncertainty was estimated using:

$u_{t}=\frac{u_{\Delta}}{\bar{x}}\cdot100$	(S8)

- Combined and expanded uncertainty

The individual components were combined by quadratic summation to obtain the combined uncertainty ($u_{c}$):

$u_{c}=\sqrt{u_{cal}^{2}+u_{r}^{2}+u_{ip}^{2}+u_{t}^{2}}$	(S9)

Finally, the expanded uncertainty ($U$) was calculated by applying a coverage factor of $k=2$, corresponding to an approximate confidence level of 95%:

$U=k\cdot u_{c}=2\cdot u_{c}$	(S10)

Table S1. Selected relevant spectral interferences for elements analyzed in hair samples by ICP-MS/MS

| Element (Isotope) | Selected spectrsocopic interferences* |
| --- | --- |
| Sodium (^23^Na^+^) | ^46^Ca^2+^ |
| Magnesium (^24^Mg^+^) | ^12^C^12^C^+^, ^48^Ca^2+^ |
| Aluminum (^27^Al^+^) | ^12^C^15^N^+^, ^13^C^14^N^+^, ^12^C^14^N^1^H^+^, ^54^Fe^2+^, ^26^Mg^1^H^+^ |
| Potassium (^39^K^+^) | ^23^Na^16^O^+^, ^38^Ar^1^H^+^ |
| Calcium (^43^Ca^+^) | ^12^C^14^N^17^O^+^, ^12^C^15^N^16^O^+^, ^13^C^14^N^16^O^+^, ^42^Ca^1^H^+^ |
| Titanium (^47^Ti^+^) | ^12^C^35^Cl^+^, ^33^S^14^N^+^, ^46^Ca^1^H^+^, ^14^N^16^O^16^O^1^H^+^, ^15^N^16^O^16^O^+^, ^31^P^16^O^+^ |
| Vanadium (^51^V^+^) | ^35^Cl^16^O^+^, ^37^Cl^14^N^+^, ^36^Ar^15^N^+^, ^36^Ar^14^N^1^H^+^, ^34^S^17^O^+^, ^33^S^18^O^+^, ^34^S^16^O^1^H^+^, ^33^S^17^O^1^H^+^, ^32^S^18^O^1^H^+^ |
| Chromium (^52^Cr^+^) | ^12^C^40^Ar^+^, ^36^Ar^16^O^+^, ^38^Ar^14^N^+^, ^35^Cl^16^O^1^H^+^, ^35^Cl^17^O^+^, ^37^Cl^14^N^1^H^+^, ^37^Cl^15^N^+^, ^36^S^16^O^+^, ^34^S^18^O^+^, ^34^S^17^O^1^H^+^, ^33^S^18^O^1^H^+^, ^40^Ca^12^C^+^, ^36^Ar^15^N^1^H^+^ |
| Manganese (^55^Mn^+^) | ^41^K^14^N^+^, ^40^Ar^15^N^+^, ^36^Ar^16^O^1^H^+^, ^38^Ar^17^O^+^, ^40^Ar^14^N^1^H^+^, ^37^Cl^16^O^1^H^1^H^+^, ^37^Cl^17^O^1^H^+^, ^23^Na^32^S^+^, ^37^Cl^18^O^+^, ^39^K^16^O^+^, ^23^Na^16^O^16^O^+^, ^54^Fe^1^H^+^, ^36^S^18^O^1^H^+^, ^38^Ar^16^O^1^H^+^, ^43^Ca^12^C^+^, ^40^Ca^15^N^+^, ^36^Ar^18^O^1^H^+^ |
| Iron (^56^Fe^+^) | ^40^Ar^16^O^+^, ^40^Ar^15^N^1^H^+^, ^38^Ar^18^O^+^, ^38^Ar^17^O^1^H^+^, ^44^Ca^12^C^+^, ^42^Ca^14^N^+^, ^40^Ca^16^O^+^, ^37^Cl^18^O^1^H^+^, ^39^K^17^O^+^, ^40^K^16^O^+^ |
| Cobalt (^59^Co^+^) | ^58^Fe^1^H^+^, ^44^Ca^15^N^+^, ^24^Mg^35^Cl^+^, ^42^Ca^16^O^1^H^+^, 42Ca17O^+^, ^43^Ca^16^O^+^, ^40^Ca^18^O^1^H^+^, ^23^Na^36^Ar^+^, ^41^K^18^O^+^, ^40^Ar^18^O^1^H^+^ |
| Nickel (^60^Ni^+^) | ^42^Ca^17^O^1^H^+^, ^46^Ca^14^N^+^, ^44^Ca^16^O^+^, ^43^Ca^17^O^+^, ^43^Ca^16^O^1^H^+^, ^42^Ca^18^O^+^, ^48^Ca^12^C^+^, ^24^Mg^36^Ar^+^, ^23^Na^37^Cl^+^, ^25^Mg^35^Cl^+^ |
| Copper (^65^Cu^+^) | ^36^Ar^14^N^14^N^1^H^+^, ^12^C^16^O^37^Cl^+^, ^48^Ca^17^O^+^, ^46^Ca^18^O^1^H^+^, ^48^Ca^16^O^1^H^+^, ^25^Mg^40^Ar^+^, ^24^Mg^40^Ar^1^H^+^, ^24^Mg^25^Mg^16^O^+^, ^23^Na^23^Na^18^O^1^H^+^, ^31^P^16^O^18^O^+^, ^31^P^17^O^17^O^+^, ^32^S^32^S^1^H^+^, ^32^S^33^S^+^, ^32^S^16^O^17^O^+^, ^32^S^16^O^16^O^1^H^+^, ^33^S^16^O^16^O^+^, ^37^Cl^14^N^14^N^+^, ^64^Zn^1^H^+^ |
| Zinc (^66^Zn^+^) | ^24^Mg^40^Ar^1^H^+^, ^48^Ca^18^O^1^H^+^, ^33^S^16^O^16^O^1^H^+^, ^65^Cu^1^H^+^, ^35^Cl^14^N^16^O^1^H^+^, ^37^Cl^12^C^16^O^1^H^+^, ^38^Ar^14^N^14^N^+^, ^26^Mg^40^Ar^+^, ^25^Mg^25^Mg^16^O^+^, ^31^P^17^O^18^O^+^, ^32^S^34^S^+^, ^33^S^33^S^+^, ^34^S^16^O^16^O^+^, ^32^S^17^O^17^O^+^, ^33^S^16^O^17^O^+^, ^32^S^16^O^18^O^+^, ^48^Ca^18^O^+^, ^48^Ca^17^O^1^H^+^ |
| Arsenic (^75^As^+^) | ^43^Ca^16^O^16^O^+^, ^35^Cl^40^Ar^+^, ^40^Ca^35^Cl^+^, ^58^Fe^16^O^1^H^+^, ^39^K^36^Ar^+^, ^36^Ar^38^Ar^1^H^+^ |
| Selenium (^78^Se^+^) | ^43^Ca^35^Cl^+^, ^38^Ar^40^Ar^+^, ^37^Cl^40^Ar^1^H^+^, ^42^Ca^36^Ar^+^, ^40^Ca^38^Ar^+^, ^40^K^38^Ar^+^ |
| Strontium (^88^Sr^+^) | ^48^Ca^40^Ar^+^, ^36^Ar^38^Ar^14^N^+^ |
| Molybdenum (^95^Mo^+^) | ^57^Fe^38^Ar^+^, ^39^K^40^Ar^16^O^+^ |
| Silver (^107^Ag^+^) | ^67^Zn^40^Ar^+^ |
| Scandium (^45^Sc^+^) | ^12^C^15^N^18^O^+^, ^13^C^15^N^17^O^+^, ^13^C^14^N^18^O^+^, ^13^C^16^O^16^O^+^, ^12^C^16^O^16^O^1^H^+^, ^14^N^14^N^16^O^1^H^+^, ^14^N^15^N^16^O^+^, ^44^Ca^1^H^+^ |
| Germanium (^72^Ge^+^) | ^38^Ar^17^O^17^O^+^, ^40^Ar^16^O^16^O^+^, ^38^Ar^16^O^18^O^+^, ^36^S^36^Ar^+^, ^36^Ar^36^Ar^+^, ^40^Ca^16^O^16^O^+^, ^56^Fe^16^O^+^, ^32^S^40^Ar^+^, ^34^S^38^Ar^+^, ^36^S^36^S^+^ |
| Rhodium (^103^Rh^+^) | ^63^Cu^40^Ar^+^, ^65^Cu^38^Ar^+^ |
| Rhenium (^185^Re^+^) | - |

*Selected spectroscopic interferences considered relevant for the analyzed isotopes and the digested hair matrix are shown. Interferences were selected from the ICP-MS spectral overlap database reported by Lomax-Vogt et al. [29], considering plausible matrix- or plasma-derived species.

Table S2. Effect of helium flow rate on signal reduction in digestion blank for selenium

| He flow rate (mL min^-1^) | Signal reduction (%) | Relative net Se sensitivity (%) | S/N |
| --- | --- | --- | --- |
| 0 | 0 | 100.0 | 10.9 |
| 2 | 40.8 | 98.1 | 24.9 |
| 3 | 80.7 | 77.6 | 29.5 |
| 4 | 96.3 | 34.1 | 18.9 |
| 5 | 99.5 | 13.3 | 23.5 |
| 6 | 99.8 | 3.5 | 16.4 |

Table S3. Recovery percentages for all measured isotopes at 5 and 200 µg kg^-1^ spike concentrations in no-gas and He-HED modes

| Isotope | No-gas | | He-KED | |
| --- | --- | --- | --- | --- |
|  | **5 µg kg^-1^** | **200 µg kg^-1^** | **5 µg kg^-1^** | **200 µg kg^-1^** |
| ^23^Na | n.a. | 113.4 | n.a. | 127.6 |
| ^24^Mg | n.a. | 127.0 | n.a. | 125.4 |
| ^27^Al | <LOQ | 165.4 | <LOQ | 154.7 |
| ^39^K | n.a. | 92.9 | n.a. | 113.1 |
| ^43^Ca | n.a. | 110.1 | n.a. | 106.4 |
| ^47^Ti | 139.3 | 91.9 | 131.8 | 100.1 |
| ^51^V | 102.6 | 106.3 | 113.4 | 113.9 |
| ^52^Cr | 79.5 | 105.7 | 98.8 | 114.9 |
| ^55^Mn | <LOQ | 20.1 | <LOQ | 108.0 |
| ^57^Fe | n.a. | 86.9 | n.a. | 104.7 |
| ^59^Co | 105.1 | 105.4 | 107.0 | 109.9 |
| ^60^Ni | 113.7 | 116.0 | 121.5 | 128.3 |
| ^65^Cu | n.a. | 110.5 | n.a. | 124.4 |
| ^66^Zn | n.a. | 84.1 | n.a. | 103.2 |
| ^75^As | 101.7 | 109.6 | 111.7 | 118.1 |
| ^78^Se | 68.4 | 123.3 | 131.6 | 129.8 |
| ^85^Rb | 125.0 | 124.3 | 128.9 | 126.6 |
| ^88^Sr | 124.6 | 119.3 | 124.0 | 120.9 |
| ^95^Mo | 82.3 | 127.6 | 109.3 | 142.0 |
| ^107^Ag | 127.6 | 110.7 | 131.7 | 117.2 |
| ^111^Cd | 119.0 | 114.0 | 122.3 | 121.4 |
| ^118^Sn | 120.8 | 120.2 | 126.7 | 128.1 |
| ^137^Ba | 118.7 | 116.2 | 115.8 | 120.2 |
| ^139^La | 120.4 | 119.4 | 118.8 | 122.6 |
| ^140^Ce | 120.5 | 120.0 | 121.0 | 124.5 |

|  | No gas | He-KED |
| --- | --- | --- |
| ⁷⁵As | 24478 | 13623 |
| ¹¹¹Cd | 28838.5 | 38201.9 |
| ⁶⁵Cu | 10784671 | 26517536 |
| ²⁰⁸Pb | 2742746 | 4792911 |
| ⁷⁸Se | 224106 | 65490.8 |
| ⁶⁶Zn | 39982864 | 42662873 |
| ⁵⁷Fe | 1120115 | 1240125 |
| ⁵⁵Mn | 1429551 | 1186312 |
| ⁸⁸Sr | 6328868 | 4393056 |
| ²⁷Al | 17208689 | 2593286 |

Figure S1. Signal for each element in no gas and He-KED modes

Signals (cps)

|  | Standards (no gas) | Samples (no gas) | Standards (He-KED) | Samples (He-KED) |
| --- | --- | --- | --- | --- |
| ⁴⁵Sc | 542002 | 1228767 | 236908 | 624783 |
| ⁷²Ge | 167722 | 261280 | 140150 | 200175 |
| ¹⁰³Rh | 983577 | 1365195 | 1654503 | 2727755 |
| ¹⁸⁵Re | 308153 | 496551 | 465276 | 935141 |

Figure S2. Signals of internal standards in calibration standards and hair samples under no gas and He-KED modes

Recovery (%)

|  | 200 mg | 100 mg | 60 mg | 20 mg |
| --- | --- | --- | --- | --- |
| As | 120.455 | 118.182 | 136.364 | 136.364 |
| Cd | 97.6 | 110.4 | 105.6 | 120.8 |
| Cu | 112.121 | 118.182 | 118.182 | 130.303 |
| Se | 95.679 | 116.667 | 110.802 | 108.333 |
| Pb | 113.084 | 114.019 | 113.551 | 137.383 |
| Zn | 112.981 | 116.346 | 115.385 | 112.981 |

Figure S3. Recoveries obtained for certified elements in ERM-DB001 at different sample masses. Recoveries were calculated relative to the certified values of the reference material. Error bars represent the expanded uncertainty of the measured concentrations propagated to the recovery values, using a coverage factor of k=2
